# Central nervous system *Aspergillus quadrilineatus* infection in a COVID‐19 patient, a case report and literature review

**DOI:** 10.1002/jcla.24971

**Published:** 2023-10-05

**Authors:** Kazem Amirizad, Mona Ghazanfari, Javad Javidnia, Mahdi Abastabar, Mohammad Taghi Haghi Ashtiani, Maryam Sotoudeh Anvari, Maryam Fathi, Amirreza Espahbodi, Hamid Badali, Mohammad Taghi Hedayati, Iman Haghani, Seyedmojtaba Seyedmousavi

**Affiliations:** ^1^ Department of Mycology, Faculty of Medical Sciences Tarbiat Modares University Tehran Iran; ^2^ Department of Medical Mycology, School of Medicine Mazandaran University of Medical Sciences Sari Mazandaran Iran; ^3^ Invasive Fungi Research Center, Communicable Diseases Institute Mazandaran University of Medical Sciences Sari Iran; ^4^ Pediatrics Center of Excellence, Children's Medical Center Tehran University of Medical Sciences Tehran Iran; ^5^ Department of Surgical and Clinical Pathology, Cardiac Research, Tehran Heart Center Tehran University of Medical Sciences; Tehran Iran; ^6^ Department of Parasitology, Faculty of Medical Sciences Tarbiat Modares University Tehran Iran; ^7^ Student Research Committee, School of Medicine Mazandaran University of Medical Sciences Sari Mazandaran Iran; ^8^ Department of Molecular Microbiology & Immunology, South Texas Center for Emerging Infectious Diseases The University of Texas at San Antonio San Antonio Texas USA; ^9^ Department of Laboratory Medicine, Microbiology Service, Clinical Center National Institutes of Health Bethesda Maryland USA

**Keywords:** acute lymphoblastic leukemia, *Aspergillus quadrilineatus*, cerebral aspergillosis, COVID‐19

## Abstract

**Background:**

Viral pneumonia such as COVID‐19‐associated aspergillosis could increase susceptibility to fungal super‐infections in critically ill patients.

**Methods:**

Here we report a pediatric case of *Aspergillus quadrilineatus* cerebral infection in a recently diagnosed COVID‐19‐positive patient underlying acute lymphocytic leukemia. Morphological, molecular methods, and sequencing were used to identify this emerging species.

**Results:**

Histopathological examination showed a granulomatous necrotic area containing dichotomously branching septate hyphae indicating a presumptive *Aspergillus* structure. The species‐level identity of isolate growing on brain biopsy culture was confirmed by PCR sequencing of the *β‐tubulin* gene as *A. quadrilineatus*. Using the CLSI M38‐A3 broth microdilution methodology, the in vitro antifungal susceptibility testing demonstrated 0.032 μg/mL MIC for posaconazole, caspofungin, and anidulafungin and 8 μg/mL against amphotericin B. A combination of intravenous liposomal amphotericin B and caspofungin therapy for 8 days did not improve the patient's condition. The patient gradually continued to deteriorate and expired.

**Conclusions:**

This is the first COVID‐19‐associated cerebral aspergillosis due to *A. quadrilineatus* in a pediatric patient with acute lymphocytic leukemia. However, comprehensive screening studies are highly recommended to evaluate its frequency and antifungal susceptibility profiles. Before being recommended as first‐line therapy in high‐risk patients, more antifungal susceptibility data are needed.

## Graphical abstract

The first COVID‐19‐associated cerebral aspergillosis was due to *Aspergillus quadrilineatus* in a pediatric patient with acute lymphocytic leukemia. A combination of intravenous liposomal amphotericin B and caspofungin therapy for 8 days, did not improve the patient's condition. The patient gradually continued to deteriorate and expired.

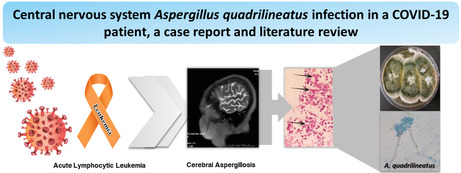

## 1 INTRODUCTION

Patients infected with severe acute respiratory syndrome coronavirus 2 (SARS‐CoV‐2) have been reported as one of the highest risk factors for opportunistic fungal infections including disseminated *Aspergillus infections*.^1^ Invasive aspergillosis (IA) is a life‐threatening fungal infection associated with high morbidity and mortality in immunocompromised patients, especially in hematopoietic stem cells and solid organ transplant recipients, neutropenic patients, and those receiving intensive chemotherapy.^2^ In contrast to pulmonary aspergillosis, cerebral *Aspergillus* infections is a rare clinical demonstration, associated with high mortality and poor outcome despite extensive antifungal therapy and surgical interventions.^1^ The incidence of cerebral aspergillosis is not clearly known and seems to be associated with the host's underlying conditions including primary immunodeficiencies.^3^ The overall prevalence is reported to be less than 7%, in patients with hematologic malignancies, and the frequency might be as high as 20%–40%.^4^, ^5^

Among genera *Aspergillus*, *A. fumigatus* followed by *A. flavus* are the primary cause of IA in individuals with COVID‐19 pulmonary infections;^6^, ^7^, ^8^ however, other aspergilli are also documented, such as *A. calidoustus*, *A. sublatus,* and *A. tubingensis*.^9^ *Aspergillus* species are one of the most ubiquitously found saprophytic molds in soil and decaying vegetation, with the potential to cause opportunistic disease primarily in patients with defective immune systems and can be divided into 13 clinically important sections.^10^ The *Nidulantes* section has been subdivided into eight series, of which there are 25 species in the *Nidulantes* portion including *A. quadrilineatus*.^11^ Overall, members of *A. nidulans* complex have been associated with chronic granulomatous disease.^10^

Here, we present a case of COVID‐19‐associated cerebral aspergillosis due to *A. quadrilineatus* in a pediatric patient with underlying acute lymphocytic leukemia (ALL).

## 2 CASE PRESENTATION

An 11‐year‐old female with no previous medical history was admitted to the emergency department of Children's Medical Center in Tehran, Iran on September 7th, 2021, demonstrating severe intermittent and generalized abdominal pain and non‐blood vomiting which prolonged for a week. There was no history of fever, diarrhea, headache, and loss of consciousness. The hematological work‐up showed pancytopenia: white blood cell count; 7.88 × 10^3^/μl (neutrophils; 13%, lymphocytes; 26%, monocytes; 5%, and blastocytes; 56%), hemoglobin; 7 mg/dL, RBC count; 2.65 × 10^6^/μ, hematocrit; 20.5% and platelet count; 32 × 10^3^/μL. The biochemical investigations demonstrated an increasing amount of uric acid; 22.7 mg/dL (children's range: 2–5.5 mg/dL), blood urea nitrogen; 60 mg/dL (1–17 years' range: 7–20 mg/dL), creatinine; 6.7 mg/dL (11 years range: 0.32–0.6 mg/dL), lactate dehydrogenase; 1800 IU/L (10–12 years' range: 120–293 U/L), high D‐dimer; 503 ng/mL (negative<350 ng/dL), elevated fibrinogen; 373 mg/dL (normal: 150–350 mg/dL), and high C‐reactive protein; 22(negative<6 mg/dL). The blood glucose, potassium, serum albumin, albumin/globulin ratio, coagulation profile, liver enzymes, urine analysis, and G6PD test were at a normal range.

The abdominal sonography showed evidence of fatty liver grade I, splenomegaly, and bilateral kidney enlargement. Based on laboratory data and persistent vomiting, patient was diagnosed with acute kidney injury, and tumor lysis syndrome was considered as the first differential diagnosis. After 12 h, the patient underwent hemodialysis, and rasburicase (0.2 mg/kg/day), allopurinol (300 mg/m^2^/day), pack cell transfusion, and platelets transfusion were conducted. On the next day of hospitalization (day 1), a bone marrow (BM) sample was collected and submitted for cytology analysis, which showed a predominant blast population in about 30% of all the analyzed cells; remarkably positive for CD20, CD19, CD22, CD34, and HLA‐DR. However, it was negative for CD3, CD10, CD17, and myeloperoxidase. B‐cell acute lymphoblastic leukemia (B ALL) was confirmed. The first abdominopelvic and chest computed tomography (CT) scan showed mild splenomegaly and a few grand glass opacities in the subpleural region of both lower lobes in favor of COVID‐19 infection which was confirmed by positive polymerase chain reaction for SARS‐CoV‐2. On the second day, the patient was moved to the COVID‐19 isolation unit and started receiving antiviral therapy through intravenous remdesivir. The treatment included a loading dose of 5 mg/kg on day 1, followed by 2.5 mg/kg every 24 h for the next 5 days. By day 6, the uric acid level had reduced to 4.5 mg/dL, which led to the discontinuation of rasburicase and allopurinol administration in addition to remdesivir treatment. Throughout the 2 weeks, the patient received supportive care, such as supplemental oxygen, noninvasive ventilation, and fluid and electrolyte support while staying in the COVID‐19 ward.

**FIGURE 1:**
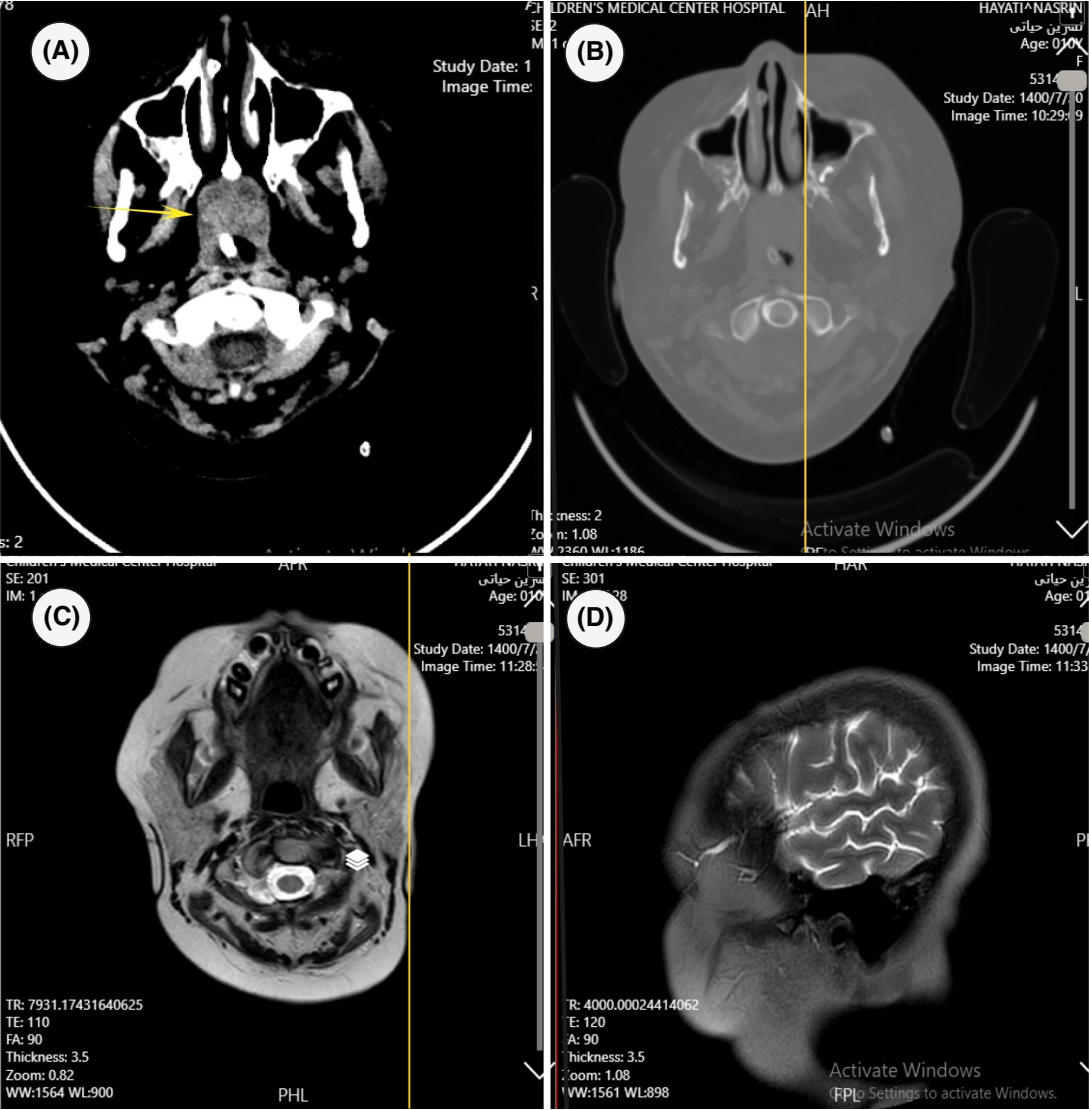
Brain MRI showing two lesions in the right parietal lobe and left frontal lobe **(**A–D).

On day 15, her COVID‐19 symptoms seemed to be resolved and recommended to initiate induction chemotherapy with L‐asparaginase, vincristine, and dexamethasone in the oncology department. Twenty‐eight days after the beginning of chemotherapy (day 43), the patient appeared lethargic and unable to speak coherent sentences. She developed bizarre movements and loss of consciousness, so immediately, she was transferred to the intensive care unit (ICU) and was intubated. The patient was re‐examined, and according to fever, petechiae, and purpura on the lower limb, she was suspected of meningococcemia; the blood culture samples were collected. Also, wide‐spectrum antibiotics (Clindamycin and Ceftriaxone) and acyclovir were initiated. The blood culture was negative. However, the brain's magnetic resonance imaging (MRI) revealed two lesions in the right parietal and left frontal lobes (Figure 1). The patient underwent left frontal osteoplastic craniotomy on day 44. The brain was edematous, and the lesion had a cortical presentation in some places. The medial side of the left frontal lobe resection was performed, and the brain biopsy sample was sent for microbiological and histopathological examination. On day 44, histopathologic hematoxylin and eosin staining demonstrated septate hyphae with dichotomous branching, resembling molds, which suspected the presence of fungal infections. Fungal culture also becomes positive 2 days after inoculating the Sabouraud dextrose agar plate (day 46), showing growth of filamentous fungi morphologically (Conidiophore, vesicle, and phialide) consistent with *Aspergillus* species.

**FIGURE 2:**
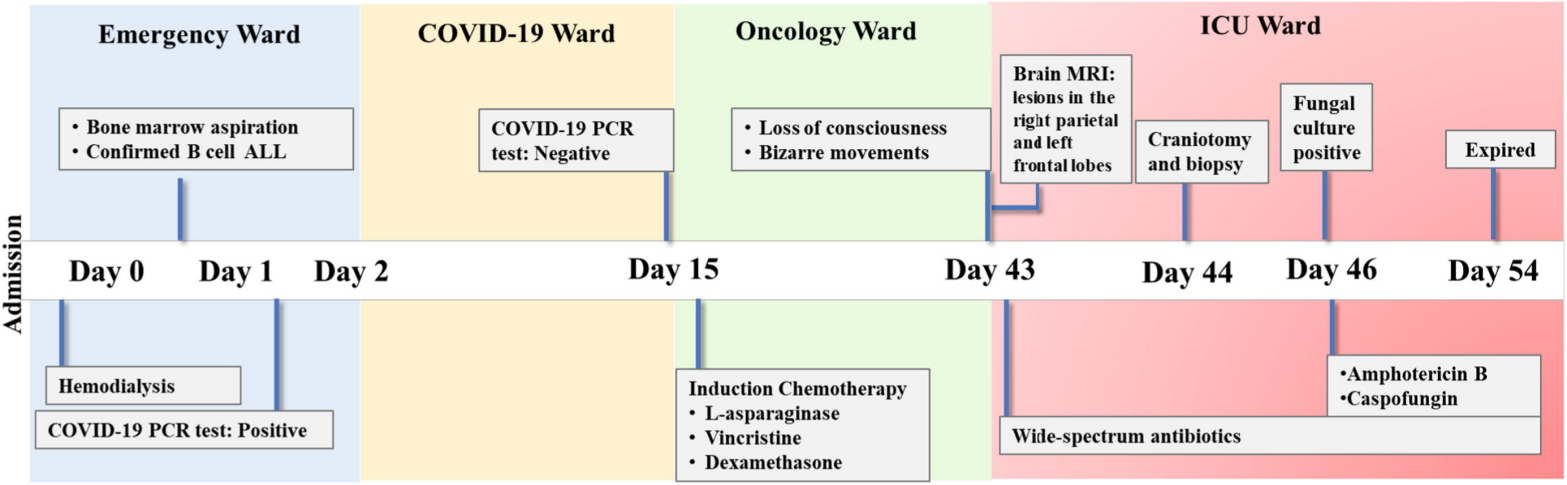
Timeline of disease progression in the course of COVID‐19 pneumonia, acute lymphocytic leukemia, and proven aspergillosis caused by *A. quadrilineatus*.

For the management of the case, antifungal therapy started with a combination of intravenous liposomal amphotericin B (5 mg/kg/day) and caspofungin (100 mg/IV/day), and concomitant antibacterial therapy (clindamycin, ceftazidime, and vancomycin). During follow‐up, a few days later, the Glasgow Coma Scale reached stage III, and there was no clinical improvement. The patient's condition continued to deteriorate and finally expired on day 54 post‐admission (Figure 2).

### 2.1 Morphological and molecular identification

**FIGURE 3:**
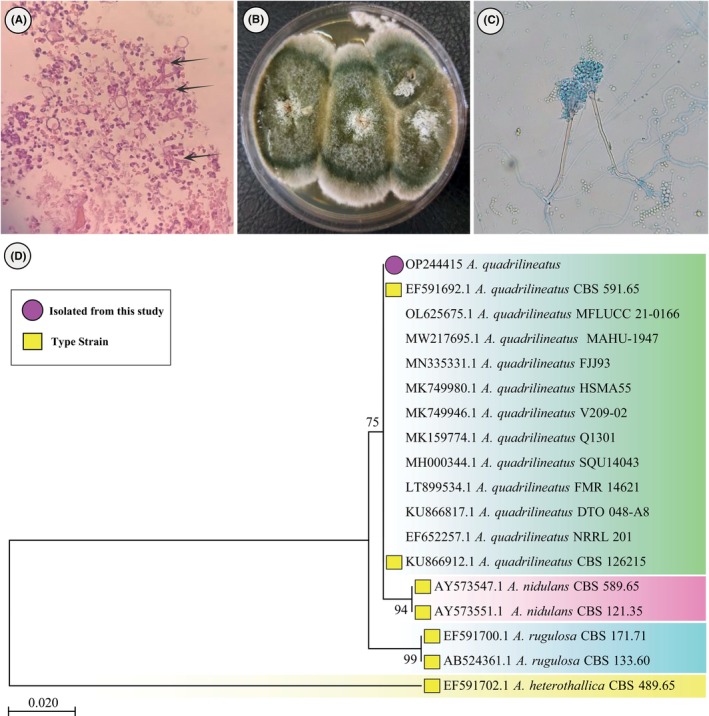
Branching septate hyphae on brain tissue biopsy using hematoxylin and eosin staining (A), *A. quadrilineatus* in sabouraud dextrose agar after 7 days growth on 37°C (B), microscopic morphology of *A. quadrilineatus*, X40 using lactophenol cotton blue (C), maximum likelihood method and Tamura‐Nei model Tree, created by MEGA‐11 from β‐tubulin sequence data of *Aspergillus* species, section *Nidulantes* using 1000 bootstrap replicates. *A. heterothallica* used as outgroup (D).

The fungal growth was maintained on SDA, and a stock culture was deposited into the Invasive Fungi Research Center (IFRC) culture collection of Communicable Diseases Institute, Mazandaran University of Medical Sciences, Sari, Iran. Macroscopic and microscopic studies were conducted and tentatively identified as *Aspergillus* species (Figure 3B,C). Morphological characteristics of *Aspergillus quadrilineatus* consist of short, columnar and biseriate conidial heads. Furthermore, conidiophore stipes are brownish, short and smooth‐walled, and conidia are rough‐walled and globose (Figure 3C). Subsequently, the isolate was sequenced using the *β‐tubulin* gene as described previously.^12^, ^13^ Obtained sequences were compared with GenBank (www.ncbi.nlm.nih.gov) and MYCOBANK database (www.mycobank.org/page/Pairwise_alignment), and isolate (accession number OP244415) was identified as *A. quadrilineatus* by showing 100% similarity with the type strains of that species (accession numbers OL625674 and AB248335), which had been initially isolated from environments. In addition, the maximum likelihood method and Tamura‐Nei model Tree, created by MEGA‐11 from β‐tubulin sequence data of *Aspergillus* species, section *Nidulantes* using 1000 bootstrap replicates. *A. heterothallica* was used as outgroup (Figure 3D).

### 2.2 Antifungal susceptibility testing

Antifungal susceptibility testing was performed according to the clinical laboratory standard institute (CLSI) guidelines M38‐A3.^14^ The minimum inhibitory concentration (MIC) in 100% inhibition of fungal growth for both azoles and amphotericin B and minimum effective concentrations (MEC) that leads to rounded compact hyphal growth compared with the unchanged growth in the control well for echinocandins were determined visually after incubating at 35°C for 48 h. The MICs for voriconazole, itraconazole, posaconazole, efinaconazole, isavuconazole, ravuconazole, amphotericin B, caspofungin, and anidulafungin against *A. quadrilineatus* were 0.25 μg/mL, 0.5 μg/mL, 0.032 μg/mL, 0.016 μg/mL, 0.032 μg/mL, 0.032 μg/mL, 8 μg/mL, 0.032 μg/mL, and 0.032 μg/mL, respectively.

### 2.3 Literature review

A comprehensive search of various databases (PubMed, Web of Science, ScienceDirect, and Scopus) was conducted to find relevant articles about fungal infections caused by *A. quadrilineatus*. The search was limited to published literature in English up to May 31, 2023. A total of 5 *A. quadrilineatus* cases that related to fungal infections were found, and details are presented in Table 1.

**TABLE 1:** Demographic characteristics, clinical data, and treatment profiles of reported cases due to *Aspergillus quadrilineatus*.

Case no.	Study	Sex/Age	Predisposing factor	Involved site	Antifungal therapy	Outcome
1	Polacheck et al.^21^	F/28	ANLL	Frontal, maxillary, and ethmoid sinusitis	AmB	Survived
2	Gugnani et al.^22^	M/60	No	Nail	Itraconazole	Survived
3	Verweij et al.^25^	M/10	CGD	Lung involvement	ND	ND
4	Sharma et al.^24^	M/45	No	Nail	Terbinafine	Survived
5	Salah et al.^23^	F/25	No	Nail	ND	Survived
6	Present case	F/11	ALL	Cerebral	Caspofungin and AmB	Died

Abbreviations: ANLL, Acute non‐lymphoblastic leukemia; CGD, Chronic granulomatosis disease; ALL, Acute lymphoblastic leukemia; AmB, Amphotericin B; ND, Not determined.

## 3 DISCUSSION

COVID‐19‐positive patients are highly susceptible to opportunistic fungal infection, including invasive candidiasis, aspergillosis, mucormycosis, and pneumocytosis due to overexpression of anti‐inflammatory cytokines, dysregulation of T‐helper cell differentiation, and impaired cell‐mediated immune response.^1^, ^6^ SARS‐CoV‐2 uses different mechanisms to suppress and compromise the human immune system like reducing T‐lymphocytes and increasing interleukins (IL‐1 and IL‐6) production.^15^ Several studies also showed that SARS‐CoV‐2, via a spike protein, binds to angiotensin‐converting enzyme 2 receptors expressed on endothelial cells of blood vessels in the brain and epithelial cells of pneumocytes, resulting in neurovascular damage and pulmonary infection, respectively.^1^ Neurovascular inflammation provides an arbitrary and suitable environment for colonizing *Aspergillus* species. Following involvement and invasion of the neurovascular system, brain abscesses, vasculitis, thrombosis, granuloma, meningitis, ventriculitis, and cerebritis occur.^16^

Cerebral aspergillosis has been primarily reported among children with ALL more frequently than acute myeloid leukemia and chronic myeloid leukemia.^17^ To the best of our knowledge, this is the first COVID‐19‐associated cerebral aspergillosis due to *A. quadrilineatus* in a pediatric patient with acute lymphocytic leukemia. The patient was in the high‐risk group because of using corticosteroids, an intravenous catheter, and broad‐spectrum antibiotics while undergoing intensive chemotherapy. These situations are essential risk factors for IFI.^18^, ^19^, ^20^ *A. quadrilineatus* belongs to the section *Nidulantes* which is phenotypically similar to *A. nidulans. Aspergillus quadrilineatus* (*Emericella quadrilineata*) is a soil fungus commonly isolated in tropical countries. *A. quadrilineatus* was a causative agent of fungal sinusitis,^21^ onychomycosis^22^, ^23^, ^24^ as well as invasive cases in CGD and leukemic patients.^21^, ^25^ Of note, these two sibling species are morphologically identical but are distinguished by molecular differences using PCR sequencing of the β‐tubulin gene.^25^ Despite the close morphologic and genetic relatedness, the two species differ significantly in their in vitro susceptibility to amphotericin B and caspofungin. However, the triazoles are active in vitro against both species.

Our study showed that amphotericin B had high MIC against *A. quadrilineatus* which is similar to previous data reported by Verweij et al. They showed that *A. quadrilineatus* was less susceptible than *A. nidulans* against caspofungin (MICs: 0.32 μg/mL), but triazoles had low MICs against both species.^25^ The earlier reports of *A. quadrilineatus* are summarized in Table 1.^21^, ^22^, ^23^, ^24^, ^25^ The proportion of males to females was 3:3, with an average of 29.8 ± 19.6 years. 33.3% of patients were children. The mortality rate was reported around 16.6%. The most infection was nail disorder (50%), followed by sinus (16.6%), lung (16.6%), and cerebral involvement (16.6%). The prognosis of cerebral aspergillosis is generally poor, regardless of the mode of therapy. Voriconazole is the first‐line regime for “proven” or “probable” invasive aspergillosis in all pediatrics as recommended by European Society of Clinical Microbiology and Infectious Diseases guidelines and ECIL‐62017 guidelines introduced isavuconazole for adults.^26^ Despite timely diagnosis and antifungal treatment, one might consider the reduced susceptibility to amphotericin B as one of the reasons leading to therapeutic failure.

The present report uses the European Organization for Research and Treatment of Cancer/Mycoses Study Group's (EORTC/MSG) proposed criteria for COVID‐19‐associated invasive aspergillosis.^27^ According to EORTC/MSG criteria, proven IFI is related to positive fungus in histology/culture of sterile tissue samples. In our study, *A. quadrilineatus* exhibited low MIC values for azoles and echinocandin agents. Similarly, in two other published reports by Salah et al. and Vidal‐Acuna et al.,^23^, ^28^ posaconazole and anidulafungin showed potent in vitro activity against *A. quadrilineatus*. Moreover, in agreement with the study conducted by Vidal‐Acuna et al.,^28^ we found that amphotericin B had high MIC value against *A. quadrilineatus*.

In summary, this case is the first clinical presentation of cerebral aspergillosis caused by *A. quadrilineatus* in a COVID‐19 patient with ALL that depicts the emerging role of this fungus in cerebral infections. With the appearance of fungal infections due to cryptic species and their possible resistance to routine antifungal agents, screening studies are highly recommended to evaluate the frequency of cryptic species, and more antifungal susceptibility data are needed before being recommended as first‐line therapy against infections due to cryptic species in high‐risk patients.

## AUTHOR CONTRIBUTIONS

All authors have made substantial contributions to the conception and acquisition of data, drafting of the article, and approval of the final version.

## FUNDING INFORMATION

This manuscript was not funded.

## CONFLICT OF INTEREST STATEMENT

Seyedmojtaba Seyedmousavi was supported by the Intramural Research Program of the National Institutes of Health, Clinical Center, Department of Laboratory Medicine. All other authors declare that there is no conflict of interest.

## Data Availability

More data is available by contacting the corresponding author.
